# Breast cystic echinococcosis with ultrasonographic “cerebral gyrus sign”: a case report

**DOI:** 10.3389/fonc.2026.1931802

**Published:** 2026-09-15

**Authors:** Na Lu, Hongping Gong, Hualin Yan, Yu Liu

**Affiliations:** 1 Department of Ultrasound, People’s Hospital of Ganzi Tibetan Autonomous Prefecture, Kangding, China; 2 Department of General Surgery, People’s Hospital of Ganzi Tibetan Autonomous Prefecture, Kangding, China; 3 Department of Medical Ultrasound, West China Hospital, Sichuan University, Chengdu, China

**Keywords:** breast mass, breast surgery, case report, cystic echinococcosis, ultrasonography

## Abstract

**Background:**

Cystic echinococcosis (CE), caused by *Echinococcus granulosus*, is a zoonotic parasitic disease that primarily affects the liver and lungs, with breast involvement being extremely rare. We report a case of breast CE in a 50-year-old Tibetan woman from an endemic area.

**Case presentation:**

A 50-year-old Tibetan woman from a pastoral area presented with a palpable mass in the left breast.Ultrasonography revealed a mixed-echoic nodule exhibiting a “cerebral gyrus sign” in the glandular layer of the left breast. MRI revealed an oval mass in the upper quadrant of the left breast, showing homogeneous high signal intensity on fat-suppressed T2-weighted images and thin peripheral ring enhancement. The final pathology diagnosis after surgery was breast CE.

**Conclusions:**

This case highlights the ultrasonographic “cerebral gyrus sign” as a potential imaging clue for breast CE and emphasizes that hydatid disease should be considered in patients from endemic regions presenting with complex breast masses, even with negative serology. Increased awareness of this rare entity may help reduce misdiagnosis and guide appropriate clinical management.

## 1 Introduction

Cystic echinococcosis caused by *Echinococcus granulosus* is a zoonotic parasitic disease that seriously threatens human health. Dogs usually serve as the definitive hosts, whereas sheep, cattle, and other ruminants act as intermediate hosts during the infectious larval stage. This uncommon zoonosis mainly occurs in endemic regions, including the Mediterranean basin, Central Asia, China, North and East Africa, Australia, and South America (1). In China, it is more frequently encountered in high-altitude pastoral areas in the western regions.

The most common form in humans is cystic echinococcosis, which is caused by the larval stage of *Echinococcus granulosus* (2). Humans are accidental hosts and become infected after ingesting parasite eggs. The embryos subsequently penetrate the intestinal wall and enter the portal venous circulation. Because the liver acts as the first filter, it is the most frequently involved organ, accounting for approximately 70% of cases. Larvae may pass through the liver and implant in the lungs, which are the second most common site, accounting for approximately 20% of cases. The remaining cases may involve the kidneys, spleen, gallbladder, pancreas, thyroid gland, brain, spinal cord, and other organs (3, 4). Breast involvement is extremely rare, accounting for only 0.27% of systemic hydatid disease (5). Ultrasonography is the preferred diagnostic modality for cystic echinococcosis. In this article, we review the literature on the etiology, risk factors, diagnosis and treatment of cystic echinococcosis and we report an exceptionally rare case of breast cystic echinococcosis.

## 2 Case presentation

A 50-year-old Tibetan woman from a pastoral area was admitted for nasal discomfort. The patient had no significant past medical history and no family history of hereditary diseases. No clinical comorbidities, such as hypertension or diabetes mellitus, were reported. During routine physical examination, a mass measuring approximately 4 × 3 cm was incidentally palpated in the upper inner quadrant of the left breast. The mass was firm, well defined, and relatively mobile. There was no tenderness, skin abnormality, or palpable enlarged lymph node. The patient had no breast-related clinical symptoms, such as pain, nipple discharge, or nipple bleeding. Routine blood tests, coagulation function, liver function, and renal function were normal. Tumor markers, including CA15–3 and carcinoembryonic antigen (CEA), were within the reference ranges.

Ultrasonography revealed a mixed-echoic nodule measuring approximately 44 × 20 × 33 mm in the glandular layer of the left breast at the 11 o’clock position, approximately 17 mm from the nipple (**Figure 1A**). The lesion was oval-shaped, with relatively well-defined margins and heterogeneous internal echogenicity. A distinctive ultrasonographic feature, characterized by a curled and whorled appearance resembling the cerebral gyri, namely, the “cerebral gyrus sign”. Color Doppler flow imaging showed no definite internal blood-flow signal within the lesion (**Figure 1B**). Ductal dilatation was observed in the right breast. No abnormally enlarged lymph nodes were detected in either axilla. The ultrasonographic impression was a mixed-echoic nodule in the left breast, categorized as BI-RADS 4A.

**Figure 1:**
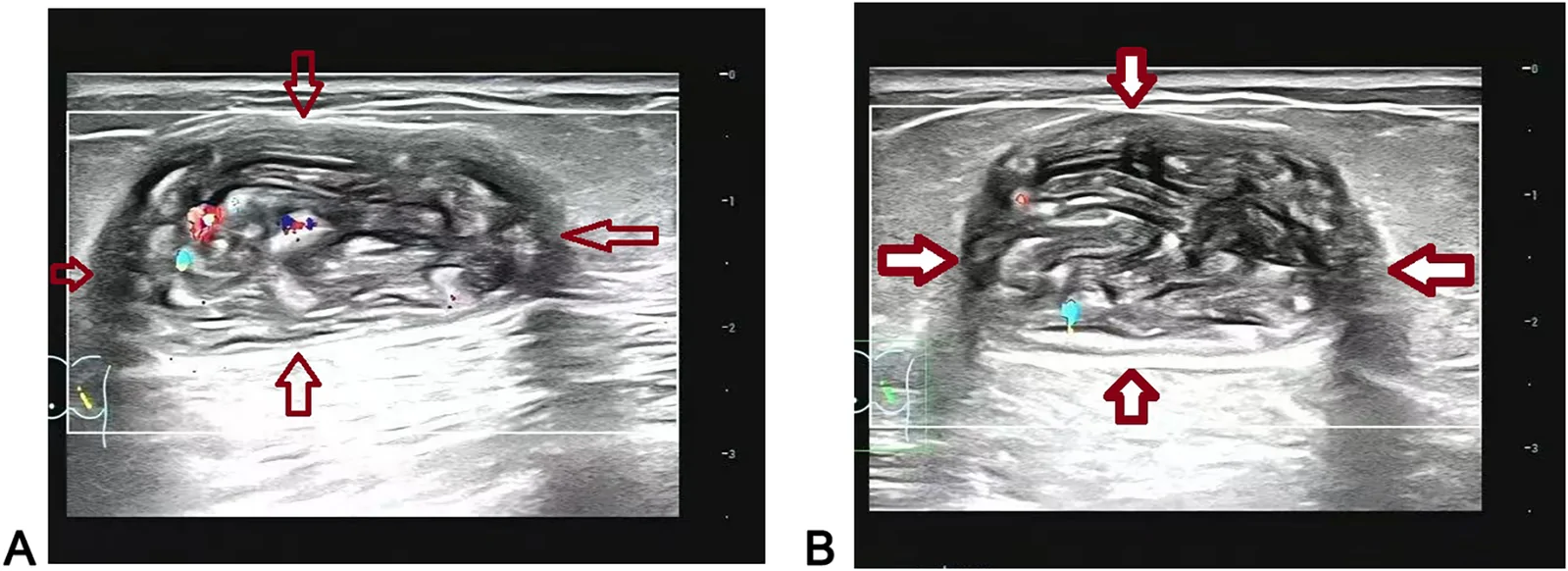
**(A)** Ultrasonographic appearance of the lesion: ultrasonography shows a mixed cystic-solid echogenic nodule in the upper inner quadrant of the left breast (arrows), with a relatively clear margin and a regular shape. **(B)** Color doppler flow imaging shows no obvious blood-flow signal within the mass (arrows).

On plain chest CT, a patchy, slightly hypodense lesion measuring 2.4 × 3.6 cm was identified in the left breast, with relatively well-defined margins. The lesion was considered a cystic mass of the breast (**Figure 2A**).

**Figure 2:**
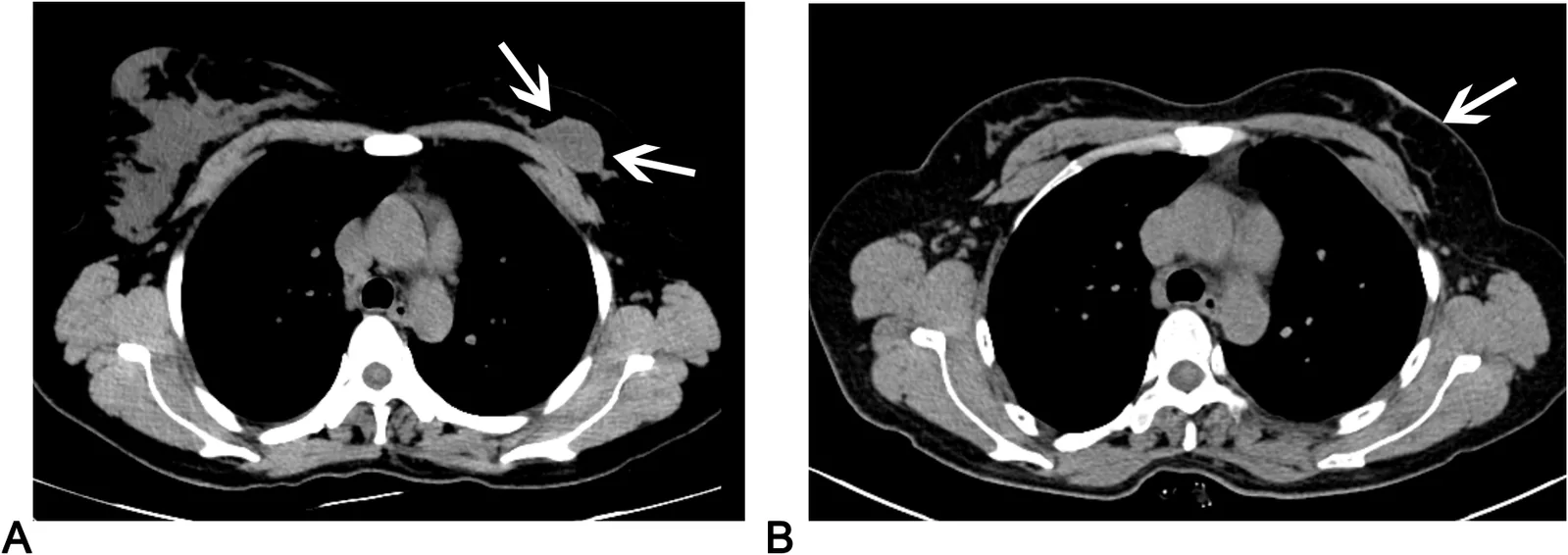
**(A)** Preoperative plain chest CT revealed a 2.4× 3.6 cm mass (arrows) in the left breast. **(B)** 40 days postoperative chest CT showed no recurrence (arrows) in the left breast.

Breast magnetic resonance imaging (MRI) showed an oval mass in the upper quadrant of the left breast on T1-weighted imaging (**Figure 3A**), measuring approximately 3.1 × 2.3 × 3.5 cm. The lesion showed homogeneous high signal intensity on fat-suppressed T2-weighted imaging (**Figure 3B**). After contrast administration, dynamic enhancement demonstrated a clear and smooth thin-walled peripheral ring enhancement, without internal enhancement (**Figure 3C**). Time-signal intensity curve analysis showed a persistent enhancement pattern. MRI suggested a mass in the upper quadrant of the left breast, most likely a benign lesion, categorized as BI-RADS 4A.

**Figure 3:**
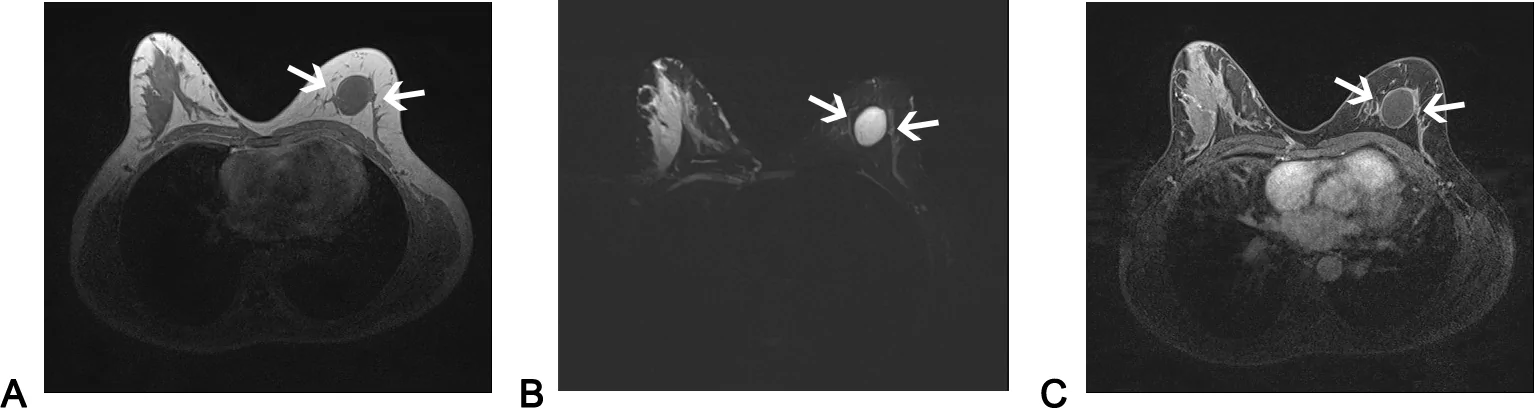
**(A)** Axial T1-weighted magnetic resonance image shows an oval mass (arrows) in the upper quadrant of the left breast. **(B)** Axial fat-suppressed T2-weighted imaging shows homogeneous high signal intensity in the lesion (arrows). **(C)** Axial fat-suppressed sequence after dynamic contrast enhancement shows clear and smooth thin-walled peripheral ring enhancement of the lesion (arrows), with no internal enhancement.

Epidemiologic investigation showed that the patient lived in an endemic area and had a long-term history of contact with dogs, cattle, and sheep. Based on laboratory findings, epidemiologic history, and imaging features, especially the peripheral ring enhancement on MRI, a benign cystic lesion was clinically suspected. Breast cystic echinococcosis was considered, although a neoplastic lesion could not be completely excluded. After multidisciplinary discussion, segmental resection of the left breast was performed.

Intraoperatively, the mass was cystic. After incision, a light-blue, translucent, preserved-egg-like endocyst membrane structure was observed (**Figure 4A**), which was consistent with the typical gross appearance of a hydatid cyst. The resected specimen was submitted for intraoperative frozen-section pathological examination, which revealed an echinococcal cyst wall in the left breast. Paraffin-embedded postoperative pathology showed homogeneous eosinophilic acellular structures arranged in parallel laminated layers, consistent with the laminated layer of a hydatid cyst, accompanied by surrounding fibrous tissue hyperplasia and chronic inflammatory cell infiltration. The final diagnosis was cystic echinococcosis (**Figure 4B**).

**Figure 4:**
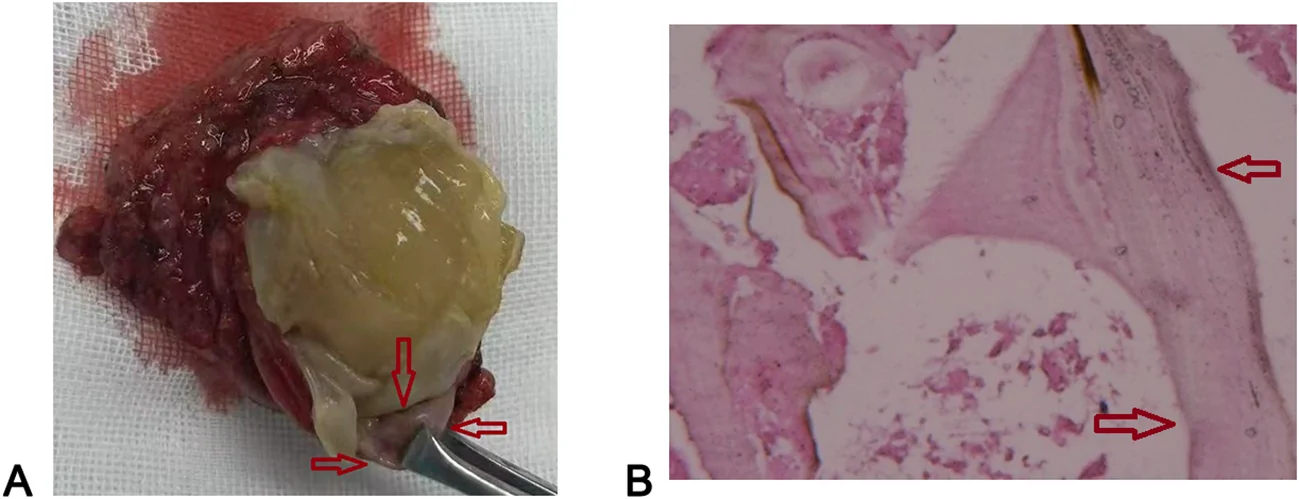
**(A)** Gross intraoperative specimen: intraoperative appearance of the breast mass showing a preserved-egg-like endocyst membrane structure (arrows); **(B)** Histopathological finding: postoperative hematoxylin and eosin staining shows homogeneous laminated endocyst components (arrows), confirming cystic echinococcosis with foreign-body giant-cell reaction.

The patient recovered well after surgery and was prescribed oral albendazole at a dose of 400 mg twice daily for 3 months after discharge, with regular follow-up recommended. At 40 days postoperatively, follow-up CT revealed no evidence of recurrence (**Figure 2B**). Meanwhile, coagulation parameters and hepatic function remained within normal limits. Telephone follow-up at 8 months after surgery revealed no abnormalities or evidence of recurrence. No adverse events, such as significant hepatotoxicity or myelosuppression, related to albendazole therapy were reported, and medication adherence was confirmed through prescription records and patient self-report. Long-term follow-up is ongoing. A timeline of the clinical course is presented in **Table 1**.

**Table 1:** Timeline of the clinical course.

Time	Clinical events
Day 0	A mass was incidentally palpated in the patient’s left breast during routine physical examination.
Day 1	Breast ultrasonography revealed a mixed-echoic nodule in the left breast, categorized as BI-RADS 4A.
Day 8	CT and CE-MRI confirmed the mass in the upper quadrant of the left breast, suggesting a benign lesion.
Day 10	Contrast-enhanced ultrasound and MRI were performed, raising suspicion of breast cystic echinococcosis.
1 day after surgery	The patient underwent surgical excision of the lesion. Intraoperative frozen-section pathological examination revealed hydatid cyst.
7 days after surgery	Histopathological examination confirmed cystic echinococcosis.
40 days after surgery	No evidence of recurrence was observed on a follow-up CT scan.
8 months after surgery	No evidence of recurrence or adverse events related to albendazole was observed during follow-up.

## 3 Discussion

The patient in this case came from an endemic area and had a definite history of animal exposure. However, serum echinococcosis testing was negative, indicating that negative serology cannot exclude the diagnosis in atypical cases. Serological testing, which detects specific antibodies against *Echinococcus* antigens, serves as an important confirmatory diagnostic method, although its sensitivity and specificity vary according to the infecting species, lesion location, and antigen used (6). Antibodies may be absent when the hydatid cyst is non-infiltrative, contains intact protoscolices, is calcified, or harbors nonviable parasites (7). Moreover, the sensitivity of serological testing decreases substantially in extrahepatic disease, with some studies reporting sensitivities below 50% (4). Therefore, a negative serology is insufficient to exclude echinococcosis. Primary hydatid disease of the breast is extremely rare, and diagnosis requires an integrated assessment of clinical history, imaging features, and pathological evidence.

Cystic echinococcosis commonly involves the liver and lungs. Reports of breast cystic echinococcosis in China and abroad are mostly individual case reports, and most cases are secondary; primary breast involvement is particularly rare. Patients initially present with a painless breast mass that gradually enlarges and usually does not involve regional lymph nodes (8). Although cases have been reported in patients aged 20 to 74 years, the most common age range is 30 to 50 years (9).

Histologically, a hydatid cyst contains three layers. The outermost fibrous host-derived layer is the pericyst. The middle layer is acellular and is referred to as the laminated membrane, whereas the innermost layer is the germinal layer, which contains the parasite and forms the endocyst. Clinically, breast hydatid cysts manifest as painless breast masses and are difficult to distinguish from fibroadenoma, simple cysts, and malignant tumors.

Several ultrasonographic signs of breast hydatid cysts have been described. The most common sign is the “double-wall sign”, in which the cyst wall appears as two echogenic layers. The “snowstorm sign” refers to the movement of hydatid sand within the lesion. In some cases, floating membranes can be observed within the lesion, resulting from detachment of the endocyst. The “congealed water-lily sign” refers to changes in the physical properties of hydatid cyst fluid; over time, the fluid becomes more viscous and the membranes eventually become immobile (10). Based on US imaging, the World Health Organization Informal Working Group on Echinococcosis (WHO-IWGE) has classified hepatic CE cysts into five types, CE1 to CE5 (11). In the present case, the “cerebral gyrus sign” was observed and was considered to result from a detached endocyst folded in a curled pattern, forming a whorled structure. This sign may represent an intermediate stage between partial and complete detachment of the endocyst in the pathogenesis of hydatid disease, which may represent the WHO CE4 type. Another hypothesis is that it reflects recent rupture of the endocyst (12). Although this sign is commonly described in hepatic cystic echinococcosis, it has rarely been reported in breast cystic echinococcosis (11).

Mammographic findings of breast cystic echinococcosis are as follows. A hydatid cyst usually appears as a homogeneous and well-circumscribed mass, sometimes accompanied by peripheral eggshell-shaped calcification, which becomes more common with a longer disease course. If the endocyst and pericyst rupture, decreased intracystic tension may lead to morphological changes, with detachment and shrinkage of the endocyst. A thin-walled cavitary appearance, known as the “annular solar eclipse sign”, may occur. In some cases, increased vascularity surrounding the cyst may lead to thickened and increased vascular signs (13).

CT offers higher anatomical resolution than US, is crucial in identifying potential complications and extrahepatic cysts, including those in the retroperitoneum, lung, mediastinum, or central nervous system. In WHO CE4 lesions, the cyst typically exhibits a solidified appearance, and a “ball-of-wool” sign can be observed due to the separation of the endocyst from the pericyst. The cystic lesion demonstrates solidification with increased, heterogeneous CT attenuation, suggestive of a solid neoplasm (7).

MRI features of breast cystic echinococcosis include the “rim sign”, characterized by a low-signal-intensity rim on T2-weighted images, which is considered characteristic of a hydatid cyst. This corresponds to the parasitic membrane and the pericyst, which is a fibrous capsule. The presence of daughter cysts produces the characteristic “cyst-within-cyst” appearance. In the early developmental stage, daughter cysts are small and round and are often arranged along the inner wall of the mother cyst in a “wheel-like” pattern. As the daughter cysts increase in number and size, they occupy the mother cyst and form a “rosette-like” structure. On T1-weighted imaging, daughter cysts show lower signal intensity than the mother cyst, and their morphology is clearly visible (13). Hydatid cysts are typically hypointense in T1W MRI and hyperintense in T2W MRI (7). Floating internal cyst membranes also show low signal intensity on all sequences and represent another characteristic imaging feature of hydatid cysts (14). Daughter cysts may appear hypointense or isointense relative to the matrix of the mother cyst on T1- and T2-weighted MR images (15). The findings of cystic lesion with capsular enhancement are suggestive of hydatid cyst. The capsular enhancement is more typical with secondary infection (15). In some cases, it may be difficult to distinguish a hydatid cyst from breast abscess or inflammatory carcinoma; therefore, clinical evaluation is essential. In the present case, contrast-enhanced imaging showed characteristic smooth, thin, peripheral ring enhancement.

Because of its rarity, hydatid disease is easily overlooked in the differential diagnosis of breast masses, even in endemic regions. This case further emphasizes that specific imaging signs, such as the “cerebral gyrus sign”, may alert radiologists to this unusual diagnosis. Mammography and MRI may also assist in diagnosis. For atypical lesions of this type, even when imaging raises concern for malignancy, percutaneous biopsy should not be performed if hydatid disease remains a possible differential diagnosis, because puncture of a hydatid lesion may cause spillage, implantation, and dissemination. When breast hydatid disease is considered preoperatively, appropriate intraoperative infection-prevention measures are also necessary.

For isolated breast hydatid cysts, complete surgical excision is the preferred and most effective treatment (16). The primary surgical goal is complete removal of the cyst without spillage, as rupture increases recurrence and systemic allergic reactions (17). Intraoperative spillage and implantation can be minimized by irrigating the cyst bed with hypertonic saline. Reported recurrence rates range from 2% to 25%, primarily due to incomplete excision or the presence of previously undetected cysts (9). For superficially located and easily accessible lesions, such as those in the breast, pericystectomy enables complete removal of cystic structures, thereby achieving definitive treatment with a low recurrence rate. Inadequate preoperative sterilization, incomplete excision, and failure to administer postoperative albendazole therapy are widely recognized as modifiable risk factors for recurrence (17).

Albendazole is the primary antiparasitic agent provided free by the Chinese government for the control and prevention of echinococcosis (18). Albendazole as the mainstay medical therapy, and is administered at 10–15 mg/kg/day in divided doses over several weeks to months. Pharmacologic treatment is indicated in three scenarios: (1) preoperative sterilization, to minimize intraoperative rupture risk, with short courses of approximately 2 weeks shown to reduce protoscolex viability; (2) postoperative prophylaxis, particularly after partial excision or spillage, with treatment commonly continued for 1–3 months after uncomplicated resection and for longer periods when spillage or residual disease is suspected; and (3) primary treatment for inoperable or disseminated cases, including CNS or bone involvement (17, 19).

The main limitation of this case is the lack of long-term follow-up data. The relatively short follow-up period of 8 months further limits the assessment of long-term outcomes, and continued monitoring is warranted to evaluate the risk of late recurrence.

## 4 Conclusion

This case report describes a cerebral-gyrus-like ultrasonographic sign in breast cystic echinococcosis, expanding the imaging spectrum of this disease and highlighting the diverse manifestations of echinococcosis. In endemic regions, clinicians and radiologists should maintain sufficient awareness of this disease. Adequate preoperative evaluation, including a detailed epidemiologic history and multimodal imaging assessment, is essential for establishing the correct preoperative diagnosis and planning appropriate surgical management to achieve complete lesion excision. The purpose of this case report is to improve awareness of this rare entity, thereby helping to avoid misdiagnosis and inappropriate management and ultimately benefiting patients.

## Data Availability

The original contributions presented in the study are included in the article/supplementary material. Further inquiries can be directed to the corresponding author.
